# One Health, One Future: A Unified Approach to a Balanced Ecosystem

**DOI:** 10.3390/tropicalmed9070164

**Published:** 2024-07-20

**Authors:** Santanu Sasidharan, Claire J. Standley

**Affiliations:** 1 Department of Physics and Astronomy, The University of British Columbia, Vancouver, BC V6T 1Z1, Canada; 2 Michael Smith Laboratories, The University of British Columbia, Vancouver, BC V6T 1Z4, Canada; 3 Center for Global Health Science and Security, Georgetown University, Washington, DC 20057, USA; 4 Heidelberg Institute of Global Health, University of Heidelberg, 69120 Heidelberg, Germany

In the past few decades, disease spillovers between humans and wildlife have increased in both frequency and severity [1,2]. The term ‘One Health’ was coined in the early 2000s, but the scientific concept itself can be traced back to the ancient Greek philosopher Aristotle, who emphasized the interdependence of human health on the ecosystem [3]. Moreover, One Health principles have been practiced for centuries, with Indigenous groups inherently integrating One Health values into their daily lives [4]. In the nineteenth century, Rudolf Virchow famously remarked that ‘Between animal and human medicine there is no dividing line-nor should there be’ [5]. The emergence of severe acute respiratory syndrome-related coronavirus 1 (SARS-CoV-1) in 2002–2003 prompted the Wildlife Conservation Society (WCS) to formulate the ‘Manhattan Principles’ in 2004, which served as the foundation for the 21st century One Health movement. These principles necessarily recognized the unpredictable nature of emerging pathogens and the need for transnational cooperation and response efforts to prevent or mitigate epidemics and pandemics [6,7]. Over the years, several efforts have been made to further understand and optimize the One Health concept, with the most recent being the One Health Joint Plan of Action (2022–2026) by the Quadripartite, a consortium that consists of the leading multilateral agencies focused on human, animal and environmental health, namely the World Health Organization (WHO), the Food and Agriculture Organization of the United Nations (FAO), the World Organisation for Animal Health (WOAH) and the United Nations Environment Program (UNEP) [8].

The WHO defines One Health as ‘an integrated, unifying approach to balance and optimize the health of people, animals and the environment’. Ironically, it has been over 20 years since the term ‘One Health’ was coined, yet the 2019 novel coronavirus (SARS-CoV-2) pandemic is a case in point which emphasizes the unmet need to study the complex interactions between humans, wildlife and the environment. With increasing globalization and urbanization, interdependence across countries is inevitable. Thus, there is a critical need for a more integrative and multidisciplinary approach to track the emergence and re-emergence of infectious diseases and to improve international health policies [9,10]. This is a call for a further understanding and implementation of the One Health approach to scientifically address and support prevention efforts.

The roots of the problem lie not only at the governmental level but also deeper, at the community or society level. Over the decades, factors such as human–animal interaction, habitat destruction, environmental pollution and changes in the climate have catalyzed the emergence and geographic spread of infectious diseases [11,12]. This Special Issue houses articles that investigate the cause and effects in various countries. A retrospective study by Hausner et al. examines the influence of socioeconomic and environmental risks, such as rainfall distribution, on the transmission of leptospirosis between dogs and humans in Brazil [13]. In contrast, Pham et al. review the advantages of the One Health approach for controlling the sporadic outbreaks of leptospirosis in Australia [14]. To further understand the impact of habitat destruction and climate changes on human–animal disease transmission, the review by Pool et al. delves into the emergence and re-emergence of cutaneous leishmaniasis in Yucatan, Mexico, emphasizing the urgent need to update the One Health strategies in the country [15]. The outcomes of the abovementioned study and reviews can be adapted and applied to other tropical countries to improve the management and detection of hotspots and transmission dynamics of infectious diseases.

While these studies focus on human–animal interactions, Ng et al. highlight the significance of molecular approaches, describing a minor variation in a conserved protein, apical membrane protein 1 of *Plasmodium knowlesi*, across different geographical regions of Malaysia and its impact on the parasite’s invasion biology [16]. The results suggest that tailored One Health measures based on the geographical origin of the parasite may be more effective than a standardized approach in the country.

This Special Issue also includes a study performed in Bangkok, Thailand, which suggests a significant risk of transmission of zoonotic hookworm *Ancylostoma ceylanicum* from semi-domesticated cats in monasteries to humans. The authors call for increased awareness and prevention measures to control human–animal transmission [17]. In addition, Nguyen et al. investigate the seroprevalence of *Toxoplasma gondii* in free-grazing ducks in integrated duck–rice production farms of Thailand [18]. The study shows the presence of the parasite in the ducks, likely due to dabbling in water contaminated with cat feces, indicating a risk of zoonotic transmission to humans through the consumption of contaminated meat. Another retrospective study in this Special Issue focuses on *Schistosoma* parasite cases in the Kingdom of Saudi Arabia and the study concludes that (i) regulating the movement of immigrants from endemic countries, (ii) monitoring the detection of the parasite in humans and animals, (iii) managing freshwater snail populations, (iv) raising public awareness and (v) developing the capacity of health professionals, veterinarians and environmental scientists are the required steps for mitigating *Schistosoma* proliferation and transmission [19]. These studies clearly illustrate how disease spillovers can occur through routine human–animal interactions in daily life. The conclusions of these studies are generally applicable to other countries in the world and provide useful insights into opportunities for improved One Health integration to manage parasitic zoonoses across diverse settings.

Another increasing concern under the One Health approach is antimicrobial resistance (AMR). AMR is a major threat to both human and animal health and its presence has been confirmed in various environmental reservoirs. An important study in this Special Issue by Pumipuntu et al. records the presence of bacterial pathogens, notably methicillin-resistant *Staphylococcus aureus* (MRSA), methicillin-susceptible *Staphylococcus aureus* (MSSA), and *Staphylococcus argenteus* in wild macaques at Kosumpee Forest Park, Thailand, raising concerns about wild macaques serving as potential carriers for the distribution of AMR in the area [20]. In another study focusing on humans, Wongsurakiat et al. find patients at a hospital in Thailand with respiratory syncytial virus (RSV)-associated acute respiratory illness, either co-infected with bacteria or suffering from bacterial superinfection, with all detected bacteria being potentially drug-resistant [21]. Similarly, the study by Helou et al. identifies RSV and *Streptococcus pneumoniae* as common etiological agents for community-acquired respiratory infections in Lebanese hospitals [22]. These authors strongly suggest that the choice of antibiotic treatment should be based on the regional AMR patterns, rapid testing, local epidemiology and targeting specific pathogens. Both studies show the need for stronger One Health surveillance to limit AMR surges through globalization and also to restrict environmental transmission to animal reservoirs.

AMR is a multidimensional problem that needs to be addressed on several levels, including policy changes. In this Special Issue, Debnath et al. perform a SWOT analysis on the AMR policies, guidelines and legislations in India and concludes that the country requires policy changes to combat AMR, such as capacity building, cost control of alternatives and incentivizing crop failures [23]. They also add that existing policies are disconnected from ground realities and need to be carefully tailored, with a focus on infrastructure and human resource development. Similarly, Intahphuak et al. conclude that the problem of antimicrobial drug resistance in Thailand can be managed by implementing a rational drug use policy, incorporating AMR surveillance through a One Health approach and educating health care workers [24]. It is noteworthy and unsurprising that the conclusions from the Indian, Lebanese and Thai studies are in agreement with the findings from the Kingdom of Saudi Arabia in terms of the need for One Health policy changes, highlighting the universality of these conclusions.

Furthermore, this Special Issue includes a protocol article for detecting emerging threats at the human–animal–environment interface, featuring a case study on the One Health surveillance system in Gujarat, India. The need for rigorous surveillance is further underscored by a study on Crimean–Congo hemorrhagic fever virus and anthrax in Armenia which highlights the necessity for strong multidisciplinary and multisectoral efforts to bolster the country’s One Health framework [25]. In Senegal, approximately 6000 km to the west of Armenia, Mhamadi et al. report the presence of Crimean–Congo hemorrhagic fever/virus in livestock, humans and ticks, emphasizing the importance of the One Health approach to prevent an outbreak of the arbovirus [26]. Meanwhile, Krokovsky et al. identify potential nosocomial *Aedes*- and *Culex*-borne transmission of arboviruses, including dengue, zika and chikungunya, which had previously been widely overlooked in surveillance and vector control programs in Brazil [27].

With contributions from various countries, this Special Issue provides a series of original research articles, reviews and protocols, featuring intriguing case studies on infectious diseases and AMR within the human–animal ecosystem. These studies aim to fill the gaps in our knowledge about the One Health approach, which is crucial for understanding and preventing emerging infectious diseases. The conclusions from these studies suggest the necessity for governmental organizations to enhance surveillance and strengthen One Health policies in various countries. Most importantly, the results provoke a critical and forward-thinking discussion, advocating a shift from traditional methods to a multidisciplinary and multisectoral approach to prepare for future epidemics and pandemics.
